# Citrus flavonoid extracts alter the profiling of rumen antibiotic resistance genes and virulence factors of dairy cows

**DOI:** 10.3389/fmicb.2023.1201262

**Published:** 2023-06-09

**Authors:** Shiqiang Yu, Liuxue Li, Huiying Zhao, Ming Liu, Linshu Jiang, Yuchao Zhao

**Affiliations:** ^1^Beijing Key Laboratory of Dairy Cow Nutrition, College of Animal Science and Technology, Beijing University of Agriculture, Beijing, China; ^2^Beijing Beinong Enterprise Management Co., Ltd., Beijing, China

**Keywords:** citrus flavone extract, dairy cow, rumen microbe, antibiotic resistance genes, virulence factors

## Abstract

Citrus flavonoid extracts (CFE) have the potential to reduce rumen inflammation, improve ruminal function, and enhance production performance in ruminants. Our previous studies have investigated the effects of CFE on the structure and function of rumen microbiota in dairy cows. However, it remains unclear whether CFE affects the prevalence of antibiotic resistance genes (ARG) and virulence factors genes (VFG) in the rumen. Therefore, metagenomics was used to identify the rumen ARG and VFG in lactating dairy cows fed with CFE diets. The results showed that CFE significantly reduced the levels of Multidrug and Antiphagocytosis in the rumen (*p* < 0.05) and increased the levels of Tetracycline, Iron uptake system, and Magnesium uptake system (*p* < 0.05). Furthermore, the changes were found to have associations with the phylum Lentisphaerae. It was concluded that CFE could be utilized as a natural plant product to regulate virulence factors and antibiotic resistance of rumen microbiota, thereby improving rumen homeostasis and the health of dairy cows.

## Introduction

Antibiotic resistance genes (ARG) are prevalent in various microbial communities, such as those inhabiting animal intestines (Looft et al., 2012), feces (Salaheen et al., 2021), soil (Perry et al., 2022), as well as rumen of ruminant livestock (Thomas et al., 2018). The differences in ARG and virulence factor genes (VFG) of microbiota significant impact animal gastrointestinal health (Wu et al., 2021). Long-term antibiotic use or misuse can lead to the evolution or acquisition of ARG, which can be transferred horizontally to pathogenic bacteria and accelerate their spread in the environment, posing a threat to the ecological and human health (Syeda et al., 2020). Additionally, ARG affects bacterial resistance, which may pose risks and disruptions to the livestock industry, affecting livestock health and causing adverse environmental impacts (Tang et al., 2019, 2021).

In ruminants, in the absence of external interference, the rumen contains many unknown ARG and VFG, which are provided by microorganisms and can affect the animal’s resistance to various external factors and play a critical role in the health of the rumen (Ma et al., 2022). The rumen of ruminants can be considered as a significant reservoir of ARG and VFG. The changes in these genes and factors are closely related to the composition of microbiota in the rumen (Ma et al., 2022). Recently, studies on rumen resistance, such as in dairy cows (Sun et al., 2021) and sheep (Yuan et al., 2022), have found that the abundance and distribution of resistome in the rumen are affected by the animals’ diet (Auffret et al., 2017). For example, Auffret et al. (2017) revealed that the dietary ratio of concentrate to forage significantly changed the ruminal ARG and VFG in beef cattle.

Nutritional strategies, such as using phytogenic feed additive have been shown to benefit the overall health and performance of ruminant livestock by regulating the balance of gastrointestinal tract microbiota (Samtiya et al., 2021). It is known that flavonoids are anti-inflammatory and anti-oxidative polyphenols, which have been shown to alter bacterial cell membrane permeability and inhibit bacterial cell wall synthesis (Song et al., 2022). Our previous study reported that feeding citrus flavonoid extracts (CFE) improved lactational performance by modulating rumen microbiota composition and function (Yu et al., 2023). However, there is limited research on the effect of natural phytochemicals on the expression of ARGs and VFGs in the rumen of dairy cows.

This study aimed to evaluate the effects of dietary CFE on ruminal ARG and VFG profiling in dairy cows. We hypothesized that citrus flavonoids could affect the expression and distribution of ARG in the rumen of dairy cows. Feeding CFE to dairy cows may affect the expression and distribution of ARG and VFG in the rumen by changing the permeability of bacterial cell membranes and inhibiting the synthesis of bacterial cell walls.

## Materials and methods

### Source of CFE

Citrus flavonoid extracts were extracted from *Citrus reticulata* Blanco and obtained from Shaanxi Xiazhou Biotechnology Co., Ltd. (Xi’an, China). The total flavonoid content of CFE was determined using commercially available kits (Beijing Solarbio Science & Technology Co., Ltd., Beijing, China), and absorbance 510 nm was recorded with rutin equivalents (Beijing Solarbio Science & Technology Co., Ltd., Beijing, China). The total flavonoid concentration of CFE was 56.83%. The three most abundant flavonoid compounds in the CFE were naringin (25.61%), hesperidin (12.89%), and neohesperidin (1.22%; Supplementary Table S1).

### Animals and treatments

The experiment was approved by the Animal Care and Use Committee of the Beijing University of Agriculture. Eight Chinese Holstein cows (662 ± 57.1 kg of body weight, 160 ± 22.4 days in milk, 36.1 ± 3.79 kg/d of milk production) were used in a replicated 4 × 4 Latin square design experiment with 25-d periods. Each experimental period consisted of a 20-d adaptation period and a 5-d sampling period. Four treatments were the basal diet (CON) and the basal diet supplemented with 50, 100, and 150 g/d CFE. The basal diets were formulated according to NRC guidelines (NRC, 2001). Cows were provided adlibitum access to total mixed ration and water. Feed ingredients and chemical composition are shown in Supplementary Table S1. Diets were offered twice daily at 0800 h and 1300 h to achieve 10% orts. Cows were milked thrice daily at 0600, 1400, and 2200 h.

### Rumen fluid collection and metagenome sequencing

On the last day of each period, approximately 200 mL of rumen fluid from each cow were collected 3 h after morning feeding using an esophageal tube. The first 150 mL of the fluid was discarded to minimize saliva contamination. Immediately after sampling, rumen fluid was filtered through a 4-layer gauze. The filtrate (50 mL) was stored in a centrifuge tube at −80°C until the DNA extraction.

Based on our previous study (Yu et al., 2023), only the rumen fluid samples from dairy cows of fed the CON and CFE at 150 g/d (CFE150) were used for metagenomic analysis. DNA extraction was performed using the standard protocol of the E.Z.N.A.^®^ Soil DNA Kit (Omega Bio-tek, Norcross, GA, USA). The genomic DNA was fragmented to an average size of 400 bp using a Covaris M220 (Gene Company Limited, China). Paired-end libraries were constructed using the NEXTFLEX Rapid DNA-Seq Kit (Bioo Scientific, Austin, TX, USA), and the pooled libraries were sequenced using the Illumina Hiseq X Ten platform (2 × 150 bp).

All data sets were subjected to quality control using Sickle (version 1.33)^1^ and Fastp (Version 0.20.0).^2^ Following quality control, the high-quality reads were aligned to the bovine genome ARSUCD1.2/bosTau9 using BWA (Version 0.7.9a).^3^ Next, the filtered reads were reassembled from scratch using Megahit (Version 1.1.2).^4^ The resulting metagenes were subjected to open reading frames (ORF) prediction using MetaGene.^5^ All predicted genes were clustered into a nonredundant gene catalog using CD-HIT (95% identity, 90% coverage).^6^ Raw sequences were mapped to predicted genes (non-redundant) using SOAP aligner (Version 2.21)^7^ to estimate their abundance.

Contigs were annotated using Diamond (Version 3.0.9)^8^ against the CARD (Version 3.0.7)^9^ and VFDB (Version 20200703).^10^ The ARG and VFG were identified by searching against a database with an E-value cut-off of E<1e^−5^ and 90% coverage, respectively. Principal coordinate analysis (PCoA) was conducted based on the Bray–Curtis dissimilarity matrix. All assembled and filtered clean raw sequence data were deposited into NCBI Sequence Read Archive under the accession number PRJNA809920.

### Statistical analysis

All statistical analyses were conducted using the R software. Permutational multivariate analysis of variance (PERMANOVA) was performed with 1,000 permutations to assess the difference in the profiles of ARG and VFG. Linear discriminant analysis (LEfSe) and the Wilcoxon rank-sum test were performed to compare the ARG and VFG between the two treatments, and significant differences were determined by an LDA score > 2 and a *p* value <0.05. We used partial least squares (PLS) for linear regression analysis of species and functions to evaluate the consistency between species and functions. The correlation analysis was conducted using Spearman’s rank correlation, and significance was determined at *p* value <0.05 and |R| > 0.5.

## Results

### The profiles of ARG and VFG

We obtained a total of 762,789,648 reads by shotgun metagenomic sequencing from 16 rumen samples. After quality control and removal of host contamination, 557,633,640 high-quality reads were obtained. The rumen metagenome comprised 93.95% bacteria, 1.47% eukaryota, 4.02% archaea, and 0.45% viruses (Supplementary Figure S1). Supplementary Table S2 displays the predominant bacterial taxa associated with ARG and VFG.

**Figure 1:**
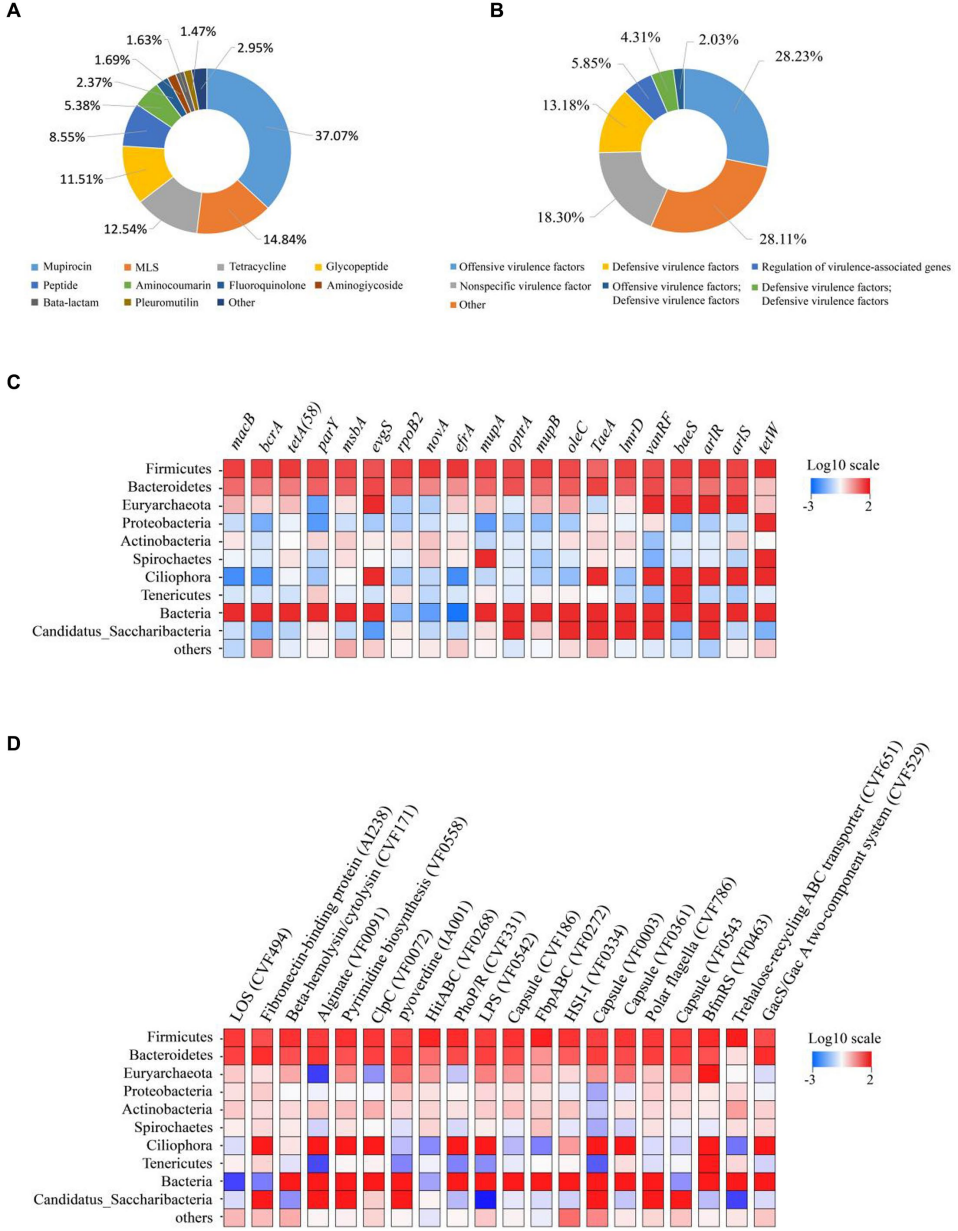
Composition of ruminal resistome and toxicity in dairy cows. **(A)** Ruminal resistome composition summarized at the antimicrobial-class level. **(B)** Ruminal toxicity composition summarized at the antimicrobial-class level-1. **(C)** ARG distributions in the phyla of anticipated rumen bacteria. The top 10 bacterial phyla are shown, with the remaining bacterial phyla included in the “others” category. The ARG distributions are depicted as colored boxes, with the top 20 resistance genes listed. **(D)** VFG distributions in the phyla of anticipated rumen bacteria. The top 10 bacterial phyla are shown, with the remaining bacterial phyla included in the “others” category. The VFG distributions are depicted as colored boxes, with the top 20 resistance genes listed.

A total of 3,880,838 contigs were annotated against the Comprehensive Antibiotic Resistance Database (CARD), identifying 523 ARGs. The identified ARGs were classified into different categories, with Mupirocin (37.07%), macrolides-lincosamids-streptogramins (MLS, 14.84%), Tetracycline (12.54%), Glycopeptide (11.51%), Peptide (8.55%), Aminocoumarin (5.38%), Fluoroquinolone (2.37%), Aminoglycoside (1.69%), Beta-lactam (1.63%), Pleuromutilin (1.47%), and other (2.95%) were found to be the most dominant categories of resistance genes (Figure 1A). The distribution of ARG was also analyzed at the class level (Supplementary Figure S2A) and Antibiotic Resistance Ontology (ARO) level (Supplementary Figure S2B) in each sample (Supplementary Figure S2). The dominant gene species and distribution were consistent across all samples. Furthermore, the distribution of the top 20 most prevalent ARG across different bacterial phyla was analyzed. It was observed that the most dominating ARG, including *macB*, *terA*, *tetA (58)*, *parY*, *msba*, and *evgs*, were dispersed across the most abundant bacterial phyla (Figure 1C).

After annotation against the Virulence Factor Database (VFDB), a total of 6,709,772 contigs were obtained, and 309 VFG were identified. The VFG were classified into offensive virulence factors (28.23%), nonspecific virulence factor (18.30%), defensive virulence factors (13.18%), regulation of virulence-associated genes (5.85%), defensive virulence factors (4.31%), offensive virulence factors (2.03%) (Figure 1B). The distribution of VFG at the level 1 (Supplementary Figure S2C) and virulence factors level (Supplementary Figure S2D) in each sample was presented in Supplementary Figure S2. Their dominant gene species and distribution had been largely consistent. When examining the distribution of the top 20 most frequently occurring VFG across different bacterial phyla, the most dominant one was present in the most abundant bacterial phyla. These VFGs include LOS (CVF494), Fibronectin-binding protein (AI238), Beta-hemolysin/cytolysin (CVF171), Alginate (VF0091), Pyrimidine biosynthesis (VF0558) and ClpC (VF0072), as displayed in Figure 1D.

### The resistance mechanism classes and types of ARG

**Figure 2:**
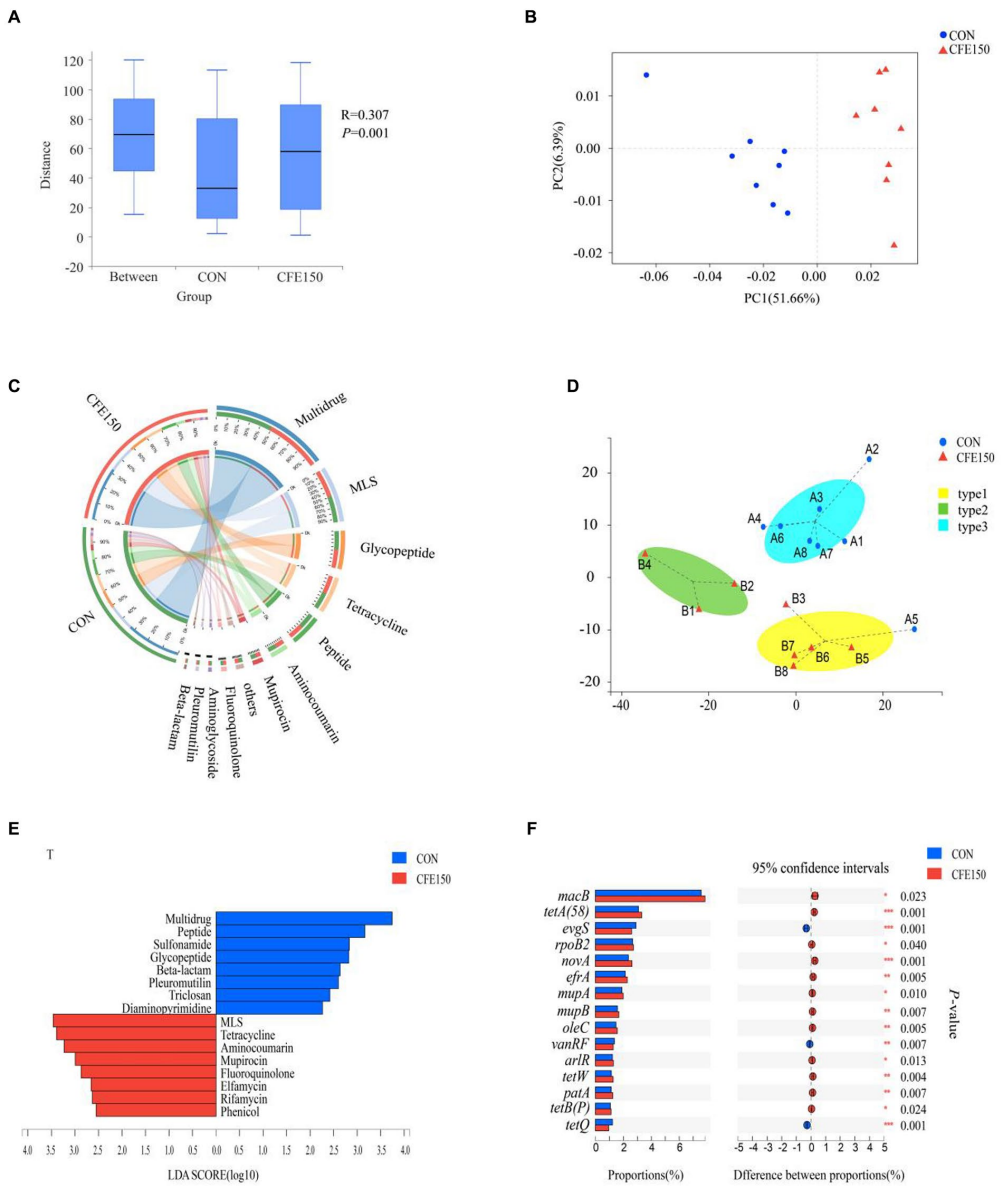
Rumen resistance profile of CFE to dairy cows. **(A)** Gene expression abundance of ARG. **(B)** Principal coordinate analysis (PCoA) of ARO levels based on Bray Curtis distance. **(C)** Composition of rumen antibiotic class level resistance function in CON and CFE150 of dairy cows. **(D)** The Genotypic assay of the ruminal resistome showed three resistance types (resistotypes) among the 16 dairy cows. **(E)** Discriminant analysis of LDA in CON and CFE150 of cows at the level of antibiotic class. **(F)** Analysis of significant differences of resistance genes between CON and CFE150 at ARO level.

Figure 2A had illustrated the effect of dietary CFE on ARG, demonstrating a significant difference in gene expression abundance between two treatments (*p* < 0.05). Additionally, at the ARO level, the PCoA plot revealed the distinct profiles of ARGs between the CON and CFE150 groups (Figure 2B). The most prevalent resistance types in the rumen at the class level were depicted in Figure 2C, and animals had been classified according to their rumen resistance types. In this study, the 16 samples were classified into three subgroups based on their rumen resistance (Figure 2D). LDA was performed on the antibiotic class level. The results showed that the eight resistance functions of Multidrug, Peptide, Sulfonamide, Glycopeptide, Beta lactam, Pleuroutilin, Triclosan, and Diaminopyrimidine were significantly enriched in the CON, while the eight resistance functions of MLS, Tetracycline, Aminocoumarin, Mupirocin, Fluoroquinolone, Elfamycin, Rifamycin, and Phenol were significantly enriched in the CFE (Figure 2E). At the antibiotic class level, Multidrum, Glycopeptide, Peptide, Beta lactam, Sulfonamide, and Trichosan were significantly enriched in the CON (*p* < 0.05), while MLS, Tetracycline, Aminocoumarin, Mupirocin, Fluoroquinolone, Phenol, Rifamycin, and Elfamycin were significantly enriched in the CFE150, consistent with the LDA discriminant analysis results (Supplementary Figure S3). Correlation analysis showed a significant correlation between ARG and diversity after CFE addition (*R*^2^ = 0.90, *p* < 0.01, Supplementary Figure S5A). Furthermore, macB, with a weighted degree of 37,393.75, was identified as playing a significant role in the analysis of both samples and the ARO functional distribution network (Supplementary Table S3; Supplementary Figure S5B). Furthermore, the expression of ARG in CON and CFE150 at the ARO level was shown in Figure 2F. It can be observed that *evgS*, *vanRF*, and *tetQ* were significantly expressed in the CON group (*p* < 0.05), whereas *MacB*, *tetA (58), rpoB2*, *novA*, *efrA*, *mupA* (conferring resistance to mupirocin) in Staphylococcus, *mupB* (conferring resistance to mupirocin) in Staphylococcus, *oleC*, *arlR*, *tetW*, *patA*, and *tetB (P)* were significantly enriched in the CFE150 group (*p* < 0.05).

### The types and mechanism of rumen virulence factors

**Figure 3:**
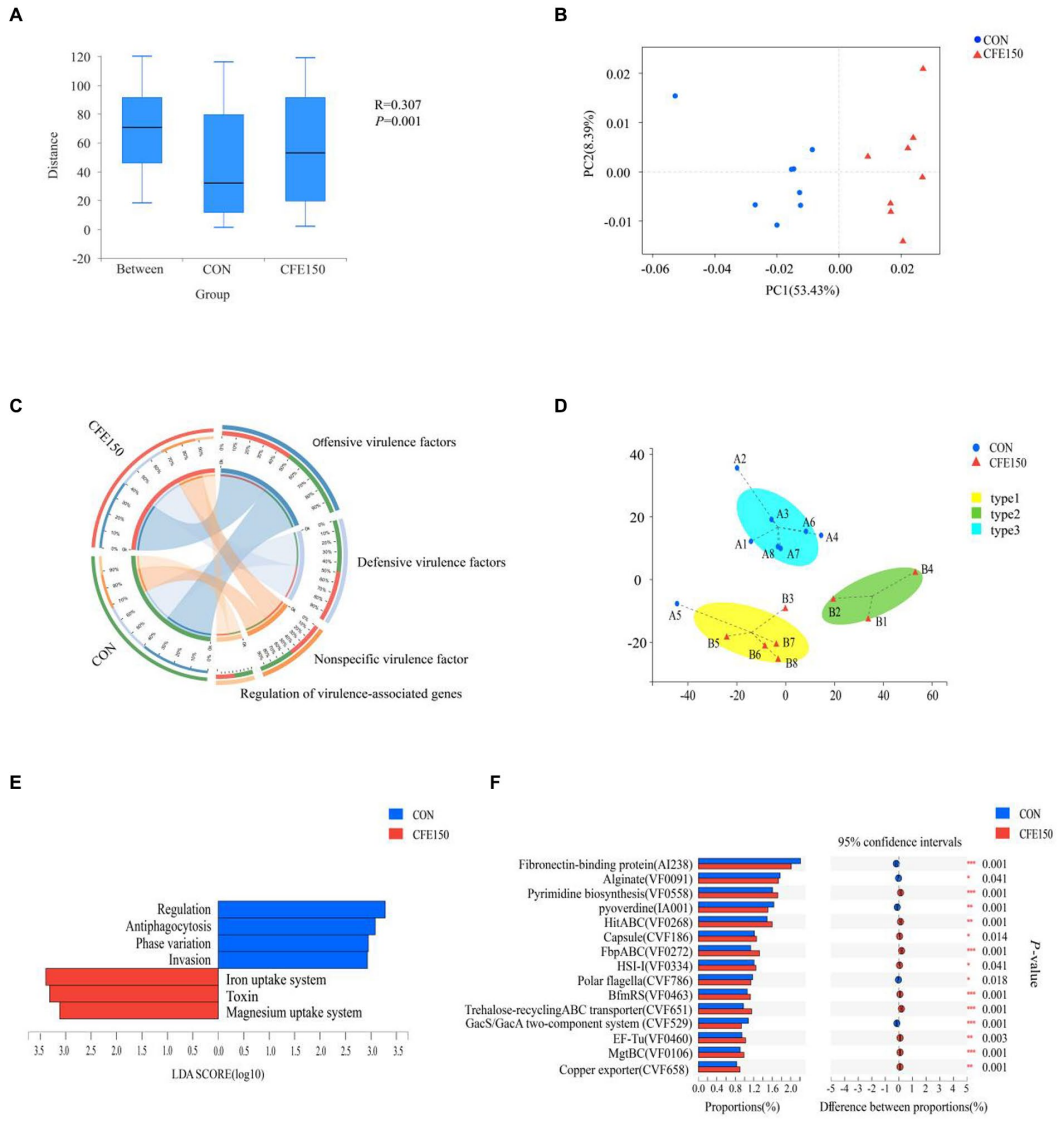
Rumen virulence profile of CFE to dairy cows. **(A)** Gene expression abundance of VFG. **(B)** Principal coordinate analysis (PCoA) of VFs based on Bray Curtis distance. **(C)** Composition of rumen VFDB level 1 virulence function in CON and CFE150 of dairy cows. **(D)** The Genotypic assay of the ruminal resistome showed three virulence types (resistotypes) among the 16 dairy cows. **(E)**: Discriminant analysis of LDA in CON and CFE150 groups of cows at the level of VFDB level 2. **(F)** Analysis of significant differences of resistance genes between CON and CFE at VF level.

The effect of CFE on VFG was shown in Figure 3A. The gene expression abundance of VFG between two treatments is significantly different (*p* < 0.05). At the virulence factors (VF) level, the samples in the PCoA plot between CON and CFE150 showed significant segregation, indicating differences between the two treatments (Figure 3B). Figure 3C illustrated the composition of the VFDB level 1, which consisted of offensive virulence factors, defensive VFG, nonspecific virulence factors, and regulation of virulence-associated genes. For this experiment, 16 samples had been categorized into three subgroups based on their rumen virulence (Figure 3D). LDA discriminant analysis was conducted on the VFDB level 2. Four toxicity functions, including regulation, antiphagocytosis, phase variation, and invasion were enriched in CON, while the CFE150 group exhibited less abundant of three toxicity functions, including iron uptake system, toxin, and magnesium uptake system compared with CON (Figure 3E). Consistent with the LDA discriminant analysis, antiphagocytosis, regulation, phase variation, and invasion were significantly enriched in the CON group (*p* < 0.05) at the VFDB level 2, while iron uptake system, toxin, and magnesium uptake system were significantly enriched in the CFE150 (*p* < 0.05) (Supplementary Figure S4). The correlation analysis showed a significant correlation between VFDB level 2 and diversity after adding CFE (*R*^2^ = 0.33, *p* = 0.021, Supplementary Figure S6A). Notably, the concentration of the iron uptake system (weighted degree = 16,4542.5) was identified as an important factor in the analysis of both samples and VF functional distribution network (Supplementary Table S4; Supplementary Figure S6B). Figure 3E displays the expression of CON and CFE150 virulence genes at VFDB level 2. Notably, fibronectin-binding protein (AI238), alginate (VF0091), pyoverdine (IA001), polar flagella (CVF786), and the GacS/GacA two-component system (CVF529) were significantly expressed in the CON group (*p* < 0.05), while pyrimidine biosynthesis (VF0558), HitABC (VF0268), capsule (CVF186), FbpABC (VF0272), HSI-I (VF0334), BfmRS (VF0463), trehalose-recycling ABC transporter (CVF651), EF-Tu (VF0460), MgtBC (VF0106), and copper exporter (CVF658) were significantly expressed in the CFE150 group (*p* < 0.05) (Figure 3F).

### Associations among rumen resistance gene, virulence factor, and bacteria in dairy cows with CFE

**Figure 4:**
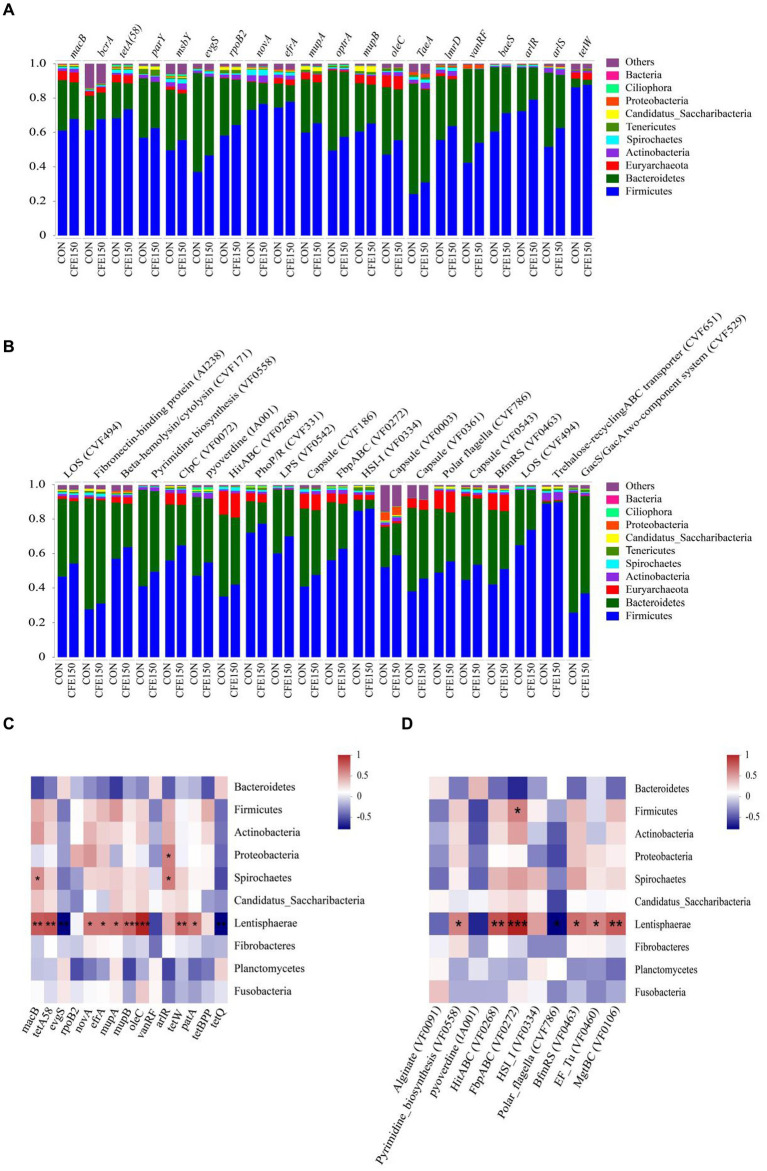
Distribution and correlation analysis of CFE on rumen bacterial ARG and VFin dairy cows. **(A)** The proportion of ARG contigs annotated to the top 10 most abundant bacterial phyla is shown in the bar plots. **(B)** The proportion of VFs contigs annotated to the top 10 most abundant bacterial phyla is shown in the bar plots. **(C)** Correlation diagram between bacteria and ARG. **(D)** Correlation diagram between bacteria and VF.

The distributions of the predominant predicted ARG (Figure 4A) and VF (Figure 4B) carrying bacterial phyla in the ruminal resistome of each group (top 20 ARG represented) had shown distinguishable patterns between the CON and CFE150. As shown in Figure 4C, Proteobacteria and Spirochaetes were positively correlated with *arlR*, while *macB* was positively correlated with Spirochaetes (*R* > 0.5, *p* < 0.05). Additionally, Lentisphaerae was positively correlated with *macB* (*R* > 0.5, *p* < 0.05), and *tetA58*, *novA*, *efrA*, *mupA*, *mupB*, *oleC*, *tetW*, *patA*, and *tetBP* were positively correlated with Lentisphaerae (*R* > 0.5, *p* < 0.05). *evgS* and *tetQ* were negatively correlated with Lentisphaerae (*R* < −0.5, *p* < 0.05). Figure 4D shows the correlation analysis results between VFs and bacteria. Lentisphaerae was found to be positively correlated with HitABC (VF0268), FbpABC (VF0272), BfmRS (VF0463), EF-Tu (VF0460), and MgtBC (VF0106) (*R* > 0.5, *p* < 0.05). Conversely, Lentisphaerae was negatively correlated with Polar flagella (CVF786) (*R* < −0.5, *p* < 0.05). Additionally, Firmicutes was positively correlated with FbpABC (VF0272) (*R* > 0.5, *p* < 0.05).

## Discussion

Previous studies have reported that natural plant products altered the profiles of ARG and VFG in the gut of poultry (Huang et al., 2018) and aquatic animals (Zhang et al., 2021). To our knowledge, this is the first study to investigate the effect of plant flavonoids on rumen ARG and VFG. In the present study, we identified a total of 523 ARG that were enriched in the rumen. These genes were classified into more than 10 different types of resistance. Similarly, Xue et al. (2021) found that the rumen resistance of cows is an individualized outcome, indicating that the rumen is a repository containing many ARG. The most prevalent ARG found in this study were *macB*, *bcrA*, *tetA (58)*, *parY*, and *masbA*. These findings align with previous research on the resistance characteristics of Spanish cow rumen, which also identified *macB* genes as the most abundant, specifically resistant to macrolides, in all of the herds (Lopez-Catalina et al., 2021). The high levels of *tetA(58)*, *tetB(P)*, *tetW*, and *tetQ* detected in this study suggest the wide prevalence of tetracycline ARGs in microorganisms, in agreement with previous research (Roberts, 2005; Tang et al., 2022). However, studies on the rumen microbiota of buffalo (Singh et al., 2012) and sheep (Yuan et al., 2022) shown different patterns of ARG abundance, indicating that there may be species-specific differences or varying dominant types due to prolonged exposure to diverse living environments and antibiotic selection pressures.

The pathogenicity of bacteria is closely linked to the expression of various cell-associated and secreted VFG, including ectoenzymes and toxins, among others (Gospodarek et al., 2009). Previous research has mainly focused on the antibiotic resistance genes and virulence factors produced by intestinal microorganisms in ruminants and their impact on the environment (Durso et al., 2011). The virulence factors produced by the rumen microorganisms may play an important role in the host’s health. In this study, the main virulence factors identified in the rumen at VFDB level 1 were offensive virus factors, non-specific virus factors, defensive virus factors, and regulation of virus-associated genes. These virulence factors are also commonly found in other host-associated microbiomes (Muhlen and Dersch, 2016), highlighting the rumen as a rich source of virulence factors.

Numerous past studies have investigated the effects of dietary components or plant-active components on the rumen microbial structure or function of dairy cows, without considering their effects on rumen resistance and virulence (Kong et al., 2010; Zhao et al., 2022a,b). Recent studies based on metagenome sequencing have shown that dietary factors can influence the microbial resistance in the feces (Liu et al., 2019) and rumen (Auffret et al., 2017) of cattle that were not exposed to antibiotics. Silva et al. (2016) reviewed the potential of plant natural products to target bacterial virulence factors. Our study further suggests that phytochemicals can affect rumen resistance genes and virulence factors in dairy cows by regulating host-microorganism-gene interactions.

Our latest research reported that CFE significantly improved milk performance by regulating the ruminal microbiota composition and function in dairy cows (Yu et al., 2023). The host–microbe interactions are crucial for the expression of ARG, colonization, and communication (Zhou et al., 2010; Capra and Laub, 2012). It has been demonstrated that tetracycline ARG are widely present in microorganisms (Roberts, 2005), including the rumen of ruminants (El and Dunlop, 2018; Jing and Yan, 2020), which is consistent with the proportion of tetracycline ARGs observed in this study. *macB*, *tetW*, *tetB (P)*, *tetQ* are the main tetracycline ARG in the rumen, and *tetW* is the most abundant gene, distributed in 28 rumen bacterial genomes, with high nucleotide sequence similarity (Sabino et al., 2019). In the present study, CFE increased the abundance of *macB*, *tetW*, *tetB (P)* and decreased the abundance of *tetQ*. Moreover, it was reported that the *tetQ* gene can encode the ribosome-protective protein that alters the ribosome’s conformation upon binding, preventing tetracycline from binding and thus conferring resistance to the antibiotic (Forsberg et al., 2014). Our study suggests that CFE may have the potential to reduce the resistance of rumen of lactating cows to tetracycline. As an important part of the rumen resistance, *macB* is a macrolide transporter that alters the resistance of strains to macrolides, such as erythromycin (Han et al., 2008; Pyörälä et al., 2014). Given that the major genera in the rumen, such as *Prevotella* and *Ruminococcus*, are carriers of ARG (Sabino et al., 2019), the CFE can also alter the abundance of *macB* by regulating rumen microbiota abundance. In the present study, we also found that Lentisphaerae was positively correlated with most of the ARG, and significantly negatively correlated with *evgS* and *tetQ*, which was directly related to the significant decline of *evgS* and *tetQ*. The primary reason for the increase or decrease in ARG following the addition of CFE may be attributed to alterations in the rumen microbiota.

ARG and VFG coexist and are interrelated, as changes in ARG can also affect virulence factors (Pan et al., 2020). It was demonstrated in our study, where CFE altered both ARG and virulence factors in the rumen. Specifically, the CFE enhanced the expression of three toxic functions at VFDB level 2, including the iron and magnesium uptake systems. It is well known that competition for iron resources occurs between hosts and pathogens, and dietary components can affect genes related to iron storage or transport (Mayneris-Perxachs et al., 2022). Bacterial pathogens recognize host cell signals in an iron-deficient environment, and weakened iron absorption can lead to disease (Skaar, 2010). Therefore, enhancing the iron uptake system can potentially improve the immune state of the host. The iron uptake system largely influences the magnesium uptake system and has beneficial effects on promoting iron absorption and inhibiting the growth of harmful bacteria (Hantke, 1997). The CFE can also weaken the antigenicity of bacteria, thereby improving the phagocytosis of bacteriophages and ultimately enhancing the immune capacity of the host (Zhe et al., 2012). The attenuation of invasion suggested that CFE may enhance organismal immunity (Clavel and Haller, 2007). Therefore, CFE enhanced rumen resistance to external stressors by altering ARG and VFG, which was essential to rumen function and host health.

Natural plant products have been shown to play a significant role in altering microbial resistance and virulence factors, as reviewed by Silva et al. (2016). Our research contributes to a deeper understanding of how natural plants can influence the microbiome of cow rumen and subsequently impact rumen antibiotic resistance and virulence. Our findings suggest that CFE can exert positive selective pressure on the rumen through the interactions between rumen microbes and genes, ultimately regulating rumen resistance and toxicity. For example, as previous studies have shown, CFE can significantly alter the abundance of Lentisphaerae, which may be an important factor leading to changes in ARG and VFG (Yu et al., 2023). The correlation analysis indicates a strong correlation between Lentisphaerae and multiple ARGs and VFGs, but the specific reasons require further investigation. However, it is important to note that rumen ecosystems are highly diverse and subject to genetic exchange and transfer through both direct and indirect mechanisms. Therefore, a more comprehensive understanding of the effects of natural plants on rumen microbiome needs more studies in the future. While our results shed light on the effects of CFE on resistance and toxicity, further research is necessary to elucidate the underlying mechanisms. This will ultimately aid in reducing the risk of rumen gene elements spreading from ruminants to the environment, and thereby minimize the risk to animal and human health.

## Conclusion

Our study revealed that the addition of 150 g/d CFE to the diet could promote rumen health by decreasing the expression of Multidrug and Antiphagocytosis genes in the rumen and increasing the expression of Iron uptake system and magnesium uptake system genes. The positive effect on rumen metabolic function of CFE indicates that citrus by-products rich in flavonoids can be used as a natural additive to regulate rumen resistance and toxicity, enhance fermentation capacity, and facilitate resource utilization and environmental protection.

## Data availability statement

The datasets presented in this study can be found in online repositories. The names of the repository/repositories and accession number(s) can be found at: https://www.ncbi.nlm.nih.gov/, PRJNA809920.

## Ethics statement

The animal study was reviewed and approved by Animal Care Committee of Beijing University of Agriculture (Beijing, China).

## Author contributions

SY and LL conducted the experiment. SY and ML collected the sample. SY and HZ analyzed the sample. YZ and LJ conceived and designed the experiments. SY and YZ wrote and revised the manuscript. All authors have read and approved the final manuscript.

## Funding

This study was supported by China Postdoctoral Science Foundation (2022M710181) and Beijing Livestock Industry Innovation Team.

## Conflict of interest

Author YZ was employed by Beijing Beinong Enterprise Management Co., Ltd.

The remaining authors declare that the research was conducted in the absence of any commercial or financial relationships that could be construed as a potential conflict of interest.

## Publisher’s note

All claims expressed in this article are solely those of the authors and do not necessarily represent those of their affiliated organizations, or those of the publisher, the editors and the reviewers. Any product that may be evaluated in this article, or claim that may be made by its manufacturer, is not guaranteed or endorsed by the publisher.

## Footnotes

^1^ https://github.com/najoshi/sickle

^2^ http://opengene.org/fastp/

^3^ http://bio-bwa.sourceforge.net

^4^ https://github.com/voutcn/megahit

^5^ http://metagene.cb.k.u-tokyo.ac.jp/

^6^ http://www.bioinformatics.org/cd-hit/

^7^ http://soap.genomics.org.cn/

^8^ http://www.diamondsearch.org/index.php

^9^ https://card.mcmaster.ca

^10^ http://www.mgc.ac.cn/VFs/main.htm
