# Supplementary material for: Citrus flavonoid extracts alter the profiling of rumen antibiotic resistance genes and virulence factors of dairy cows
Source: Front Microbiol. 2023 Jun 9;14:1201262. doi: 10.3389/fmicb.2023.1201262 (PMC10289158; doi:10.3389/fmicb.2023.1201262)
Supplement: Supplementary file 1 [file Data_Sheet_1.docx]

**Table S1** Ingredients and chemical composition of the experimental diet

| **Item** | **Contents** |
| --- | --- |
| Ingredient (% of DM) | |
| Corn silage | 34.59 |
| Alfalfa hay | 11.85 |
| Oat hay | 4.33 |
| Ground Corn grain | 17.73 |
| Flaked corn | 3.98 |
| Soybean meal | 10.46 |
| Canola meal | 2.90 |
| Sugar beet pulp | 0.56 |
| Whole cottonseed | 7.12 |
| Fat powder^2^ | 1.33 |
| Mineral and vitamin mix^2^ | 3.87 |
| Molasses | 1.28 |
| Chemical composition (% of DM)^3^ |  |
| DM, % as fed | 51.43 |
| OM | 91.12 |
| CP | 17.03 |
| NDF | 33.85 |
| ADF | 16.70 |
| EE | 4.96 |
| Starch | 25.69 |
| NE_L_ (Mcal/kg of DM)^4^ | 1.66 |

^1^Bergafat, a saturated free fatty acid supplement (Berg+Schmidt GmbH & Co. KG, Hamburg, Germany)

^2^Formulated to contain (as-is basis) trace mineral mix, 0.95%; dry corn distillers grains with solubles, 44.9%; MgO (56% Mg), 7.8%; NaCl, 6.6%; vitamin ADE premix, 0.48%; limestone, 39.2%; and selenium premix, 0.07%. Ca, 14.3%; P, 0.38%; Mg, 4.57%; K, 0.46%; S, 0.39%; Zn, 1,218 mg/kg; Fe, 186 mg/ kg, Se, 6.93 mg/kg; Cu, 370 mg/kg; vitamin A, 272,000 IU/kg; vitamin D, 75,000 IU/kg; and vitamin E, 2,080 IU/kg

^3^Analyzed values

^4^Calculated according to NRC (2001)

**Table S2** The relative abundance of the main ARGs and VFGs of the predicted bacterial genera

| Genera | Proportion | standard deviation | Coefficient of variance |
| --- | --- | --- | --- |
| Prevotella | 20.81% | 0.23% | 0.24% |
| unclassified_o__Clostridiales | 11.43% | 0.11% | 0.11% |
| unclassified_f__Lachnospiraceae | 10.00% | 0.10% | 0.11% |
| unclassified_d__Bacteria | 6.86% | 0.04% | 0.04% |
| unclassified_f__Ruminococcaceae | 5.52% | 0.03% | 0.03% |
| unclassified_o__Bacteroidales | 3.60% | 0.03% | 0.03% |
| unclassified_f__Erysipelotrichaceae | 2.42% | 0.03% | 0.03% |
| Sarcina | 2.25% | 0.03% | 0.03% |
| unclassified_p__Firmicutes | 2.42% | 0.02% | 0.02% |
| Stomatobaculum | 1.99% | 0.02% | 0.02% |
| Butyrivibrio | 1.89% | 0.02% | 0.02% |
| unclassified_f__Rikenellaceae | 1.83% | 0.02% | 0.02% |
| Bacteroides | 1.82% | 0.02% | 0.02% |
| unclassified_f__Prevotellaceae | 1.33% | 0.02% | 0.02% |
| Olsenella | 0.98% | 0.02% | 0.02% |
| Ruminococcus | 1.52% | 0.01% | 0.01% |
| unclassified_c__Clostridia | 1.27% | 0.01% | 0.01% |
| unclassified_d__unclassified | 1.11% | 0.01% | 0.01% |
| Faecalibacterium | 1.04% | 0.01% | 0.01% |
| Succiniclasticum | 0.97% | 0.01% | 0.01% |
| unclassified_f__Porphyromonadaceae | 0.87% | 0.01% | 0.01% |
| Alistipes | 0.75% | 0.01% | 0.01% |
| Eubacterium | 0.68% | 0.01% | 0.01% |
| Clostridium | 0.66% | 0.01% | 0.01% |
| Treponema | 0.54% | 0.01% | 0.01% |
| unclassified_f__Clostridiaceae | 0.45% | 0.01% | 0.01% |
| Lachnoclostridium | 0.45% | 0.01% | 0.01% |
| Stylonychia | 0.34% | 0.01% | 0.01% |
| unclassified_o__Coriobacteriales | 0.26% | 0.00% | 0.01% |
| other | 13.94% | 0.08% | 0.08% |

**Table S3** Network node properties of samples and AROs

| Type | Node Name | Degree | Weighted Degree |
| --- | --- | --- | --- |
| 1 | macB | 2 | 37393.75 |
| 1 | bcrA | 2 | 21575 |
| 1 | tetA(58) | 2 | 15529 |
| 1 | Streptomyces rishiriensis parY mutant conferring resistance to aminocoumarin | 2 | 13994.75 |
| 1 | msbA | 2 | 13360 |
| 1 | evgS | 2 | 13320.75 |
| 1 | rpoB2 | 2 | 13066.25 |
| 1 | novA | 2 | 12110.5 |
| 1 | efrA | 2 | 10729.5 |
| 1 | Staphylococcus mupA conferring resistance to mupirocin | 2 | 9455.75 |

**Table S4** Network node properties of samples and VFs

| Type | Node Name | Degree | Weighted Degree |
| --- | --- | --- | --- |
| 1 | Iron uptake system | 2 | 164542.5 |
| 1 | Adherence | 2 | 138588.25 |
| 1 | Antiphagocytosis | 2 | 136518.25 |
| 1 | Secretion system | 2 | 110819.5 |
| 1 | Regulation | 2 | 92617.75 |
| 1 | Toxin | 2 | 85753.5 |
| 1 | Serum resistance | 2 | 44702 |
| 1 | Stress protein | 2 | 36983.25 |
| 1 | Phase variation | 2 | 17742.25 |
| 1 | Invasion | 2 | 15727.75 |

**
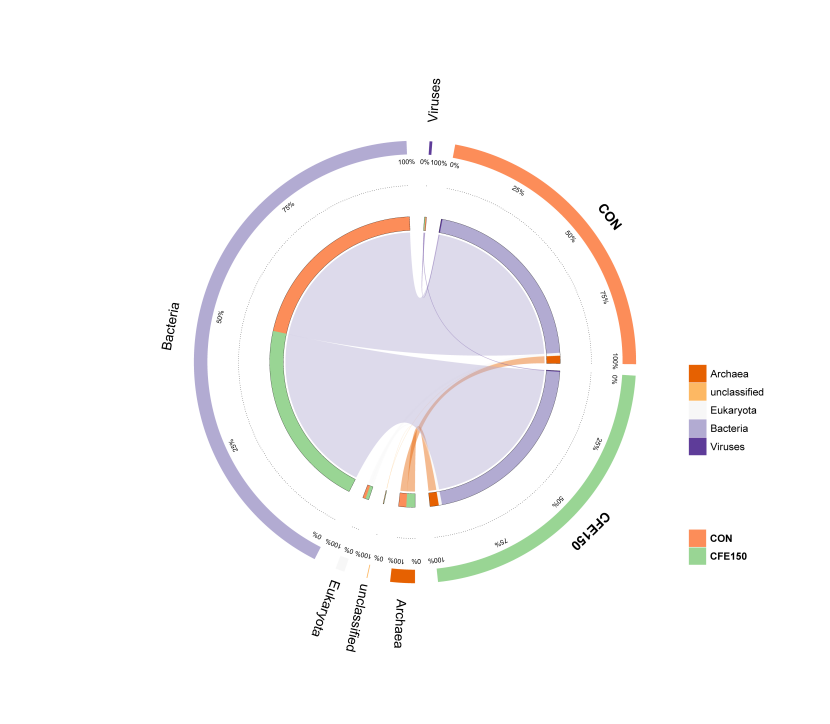
**

**Fig. S1** Composition of the rumen microbes at the domain level between CON and CFE150 samples

**
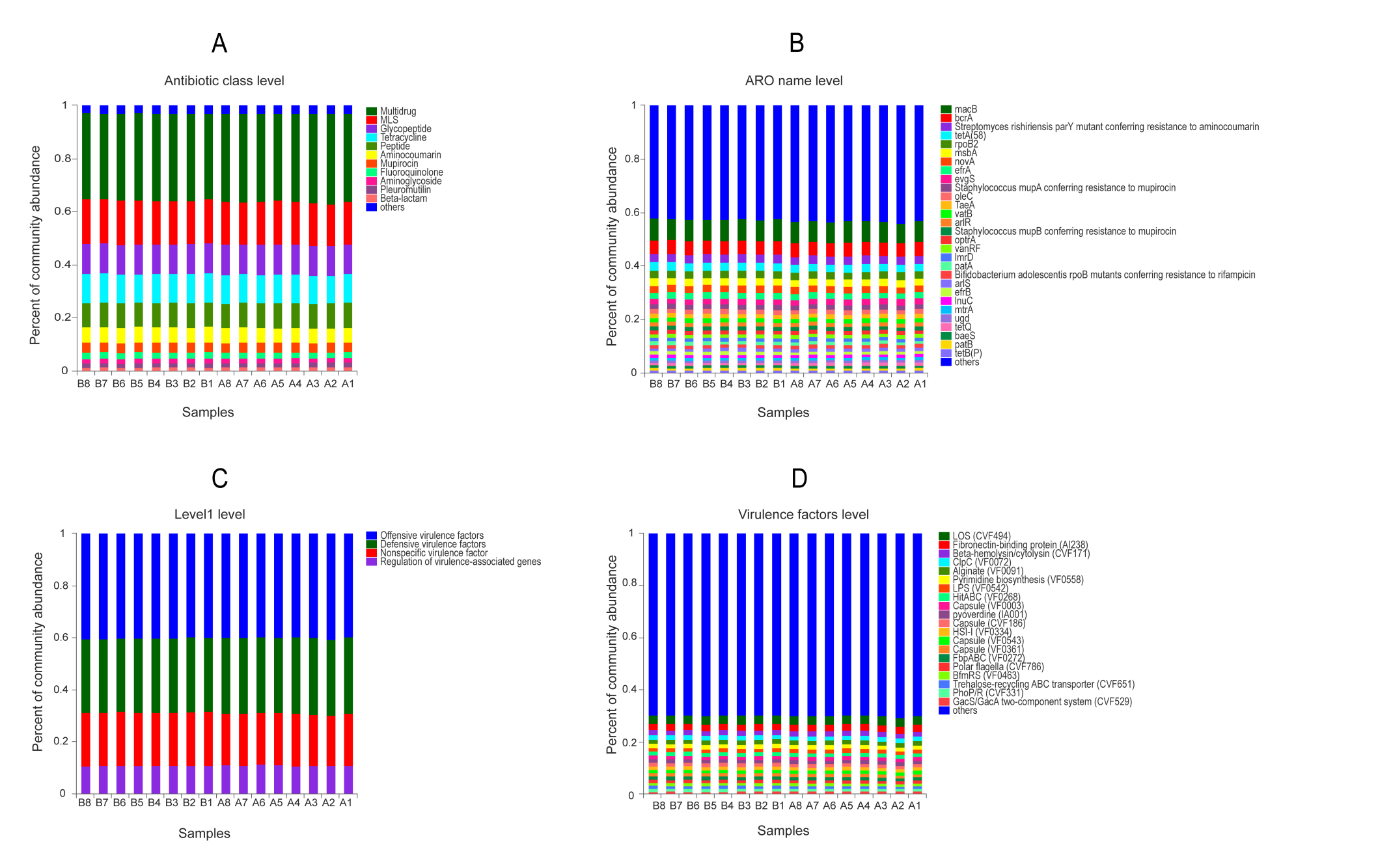
**

**Fig. S2** Composition of resistance function and toxicity of 16 dairy cows.A: Resistance composition at the antibiotic class level. B: Resistance composition at the ARO level. C: Virulence composition at level 1. D: Virulence composition at virulence factors level.

**
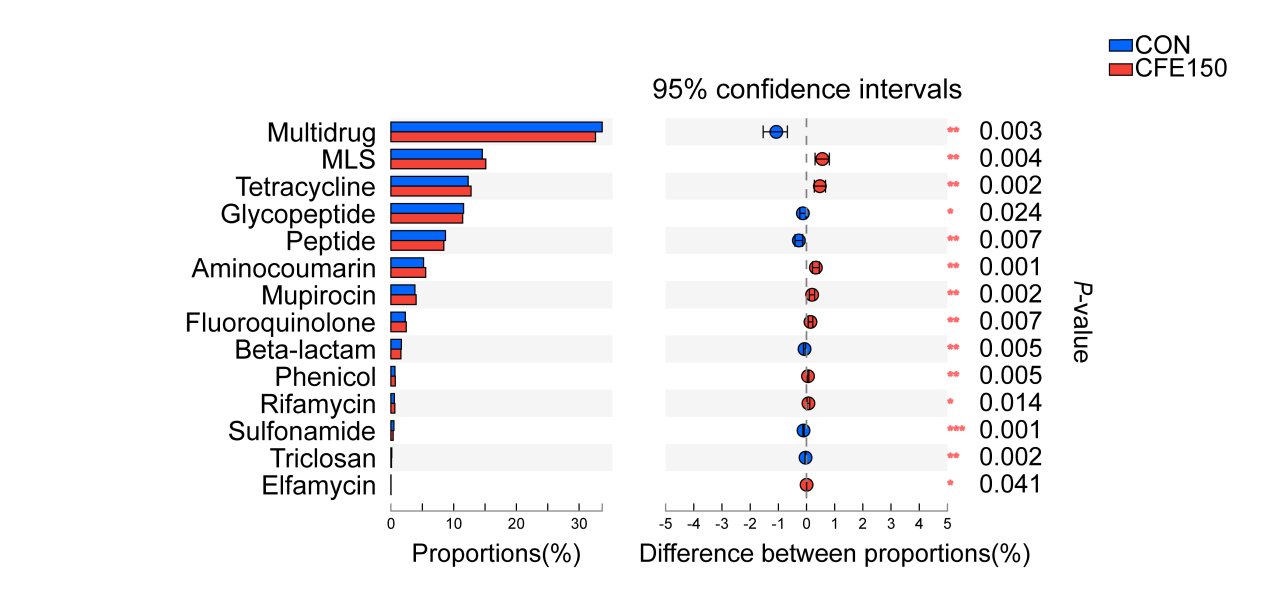
Fig. S3** Difference between CON and CFE150 at antibiotic class level


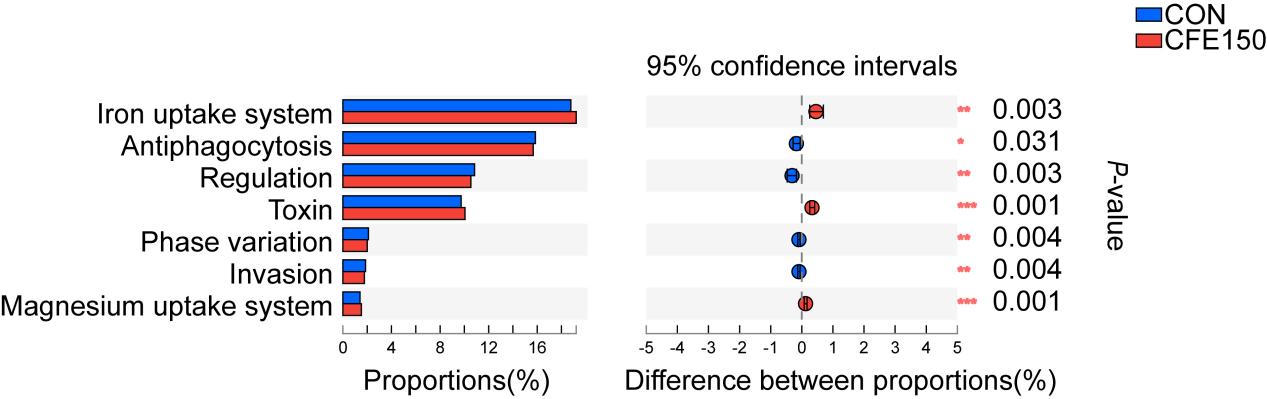


**Fig. S4** Difference between CON and CFE150 at VFDB level 2.


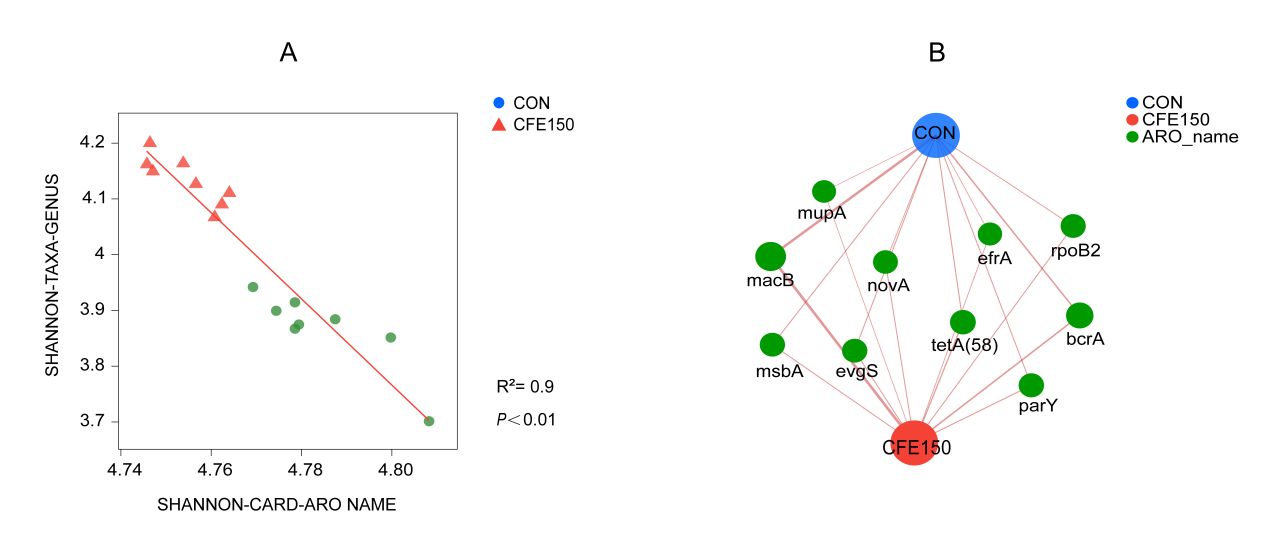


**Fig. S5** Regression analysis of species and functions of ARGs (A) and network analysis of species functional redundancy (B).

**
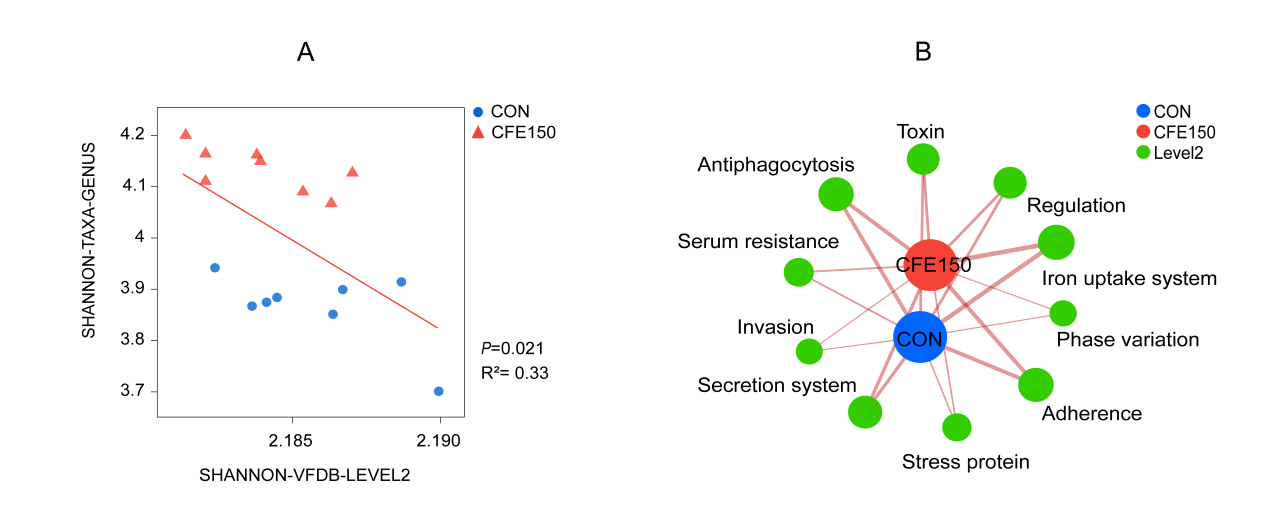
**

**Fig. S6** Regression analysis of species and functions of VFGs (A) and network analysis of species functional redundancy (B).
